# Supplementary material for: Mitigating carbon footprint for knowledge distillation based deep learning model compression
Source: PLoS One. 2023 May 15;18(5):e0285668. doi: 10.1371/journal.pone.0285668 (PMC10184906; doi:10.1371/journal.pone.0285668)
Supplement: Attachment — Submitted filename: Response.pdf [file pone.0285668.s001.pdf]

March 29, 2023

Editor-in-Chief
PLOS ONE

Dear Editor-in-Chief,

Thank you for allowing us to submit a revised draft of the manuscript PONE-D-23-01498 entitled "Mitigating Carbon Footprint for Knowledge Distillation Based Deep Learning Model Compression" for publication in the journal PLOS ONE. We appreciate the time and effort you and the reviewers dedicated to providing feedback on our manuscript. We are grateful for the insightful comments, and valuable improvements suggested for our paper. We have addressed all the questions and comments raised by the reviewers and revised the manuscript accordingly. Please find our responses to the reviewers' comments in the following pages, which contain additions and corrections made in the manuscript. All page numbers refer to the revised manuscript file with tracked changes.

Thank you very much for your consideration of this manuscript.

Sincerely,

Dr. Shafin Rahman (Corresponding Author)
Assistant Professor
Department of Electrical and Computer Engineering
North South University, Dhaka, Bangladesh
E-mail: shafin.rahman@northsouth.edu

Response to The Editorial Board

Academic Editor's Comments

1. Please ensure that your manuscript meets PLOS ONE's style requirements, including those for file naming.

   Response:
   We ensure the manuscript maintains the PLOS ONE style requirements.

2. We note that you have stated that you will provide repository information for your data at acceptance. Should your manuscript be accepted for publication, we will hold it until you provide the relevant accession numbers or DOIs necessary to access your data. If you wish to make changes to your Data Availability statement, please describe these changes in your cover letter and we will update your Data Availability statement to reflect the information you provide.

   Response:

   Regarding the Data Availability statement, we selected "Yes - all data are fully available without restriction". We are sorry to create any confusion in this regard. To clarify, we did not collect any new data sets for the experiments, rather all data sets used are publicly available from different sources and are popular within the Computer Vision literature. We would be obliged if the following can be accepted as our **Data Availability Statement**.

   "All code to reproduce the results are available from `https://github.com/King-Rafat/STKD_CFMitigation`
   The data underlying the results presented in the study are available from `https://www.cs.toronto.edu/~kriz/cifar-10-python.tar.gz`,
   `https://www.cs.toronto.edu/~kriz/cifar-100-python.tar.gz`,
   `http://cs231n.stanford.edu/tiny-imagenet-200.zip`,
   `http://host.robots.ox.ac.uk/pascal/VOC/voc2012/VOCtrainval_11-May-2012.tar`,
   `http://host.robots.ox.ac.uk/pascal/VOC/voc2007/VOCtrainval_06-Nov-2007.tar`, and
   `http://host.robots.ox.ac.uk/pascal/VOC/voc2007/VOCtest_06-Nov-2007.tar`"

   More information on each Data set is given below for further clarification if required.

   CIFAR 10

   website: `https://www.cs.toronto.edu/~kriz/cifar.html`

   download link: `https://www.cs.toronto.edu/~kriz/cifar-10-python.tar.gz`

   License type: MIT license

CIFAR 100

website: `https://www.cs.toronto.edu/~kriz/cifar.html`

download link: `https://www.cs.toronto.edu/~kriz/cifar-100-python.tar.gz`

License type: MIT license

Tiny ImageNet

website: `http://www.image-net.org/download-images`

download link: `http://cs231n.stanford.edu/tiny-imagenet-200.zip`

License type: MIT license

PASCAL VOC 2012

website: `http://host.robots.ox.ac.uk/pascal/VOC/`

download link (Train Set): `http://host.robots.ox.ac.uk/pascal/VOC/voc2012/VOCtrainval_11-May-2012.tar`

PASCAL VOC 2007

website: `http://host.robots.ox.ac.uk/pascal/VOC/`

download link (Train Set): `http://host.robots.ox.ac.uk/pascal/VOC/voc2007/VOCtrainval_06-Nov-2007.tar`

download link (Test Set): `http://host.robots.ox.ac.uk/pascal/VOC/voc2007/VOCtest_06-Nov-2007.tar`

3. We note that Figure 2 in your submission contain copyrighted images. All PLOS content is published under the Creative Commons Attribution License (CC BY 4.0), which means that the manuscript, images, and Supporting Information files will be freely available online, and any third party is permitted to access, download, copy, distribute, and use these materials in any way, even commercially, with proper attribution.

   Response:
   Thanks for pointing this out. Now, we have removed the copyrighted image. Instead, we have used new images for Figure 2, from the CIFAR 10 dataset under the MIT license. This license allows the data (images) to be published. Details about the license of the CIFAR 10 dataset can be found here: `https://github.com/wichtounet/cifar-10/blob/master/LICENSE`.

Note to The Reviewers

1. All modified portions in the revised manuscript are described in the blue-colored text.
2. In answering the comments, we have used the following format throughout this response letter. We first state the comments made by the respective reviewer, followed by our response. We then provide the changes made in the revised manuscript to address these comments. These changes are provided within the confines of a bounding box.

[Tribute] We would like to pay our highest level of gratitude to the anonymous reviewers for their very insightful comments that helped us a lot to enrich the quality of the paper. We have tried our best to incorporate the suggestions demonstrating the effectiveness of our work.

Response to The Reviewers

Responses to the review comments are illustrated below:

Reviewer 1

Reviewer Comment 1.1 —

Knowledge distillation based methods are very important nowadays due to providing an efficient computation for small hardware resources. The paper is written in this direction. In general, I like the paper including theoretical background and experimental results. Results are clear to prove their hypothesis. On the other hand, it would be better if the following revisions are applied in the next version of the manuscript.

Response:

Thank you for your encouraging comments regarding our work. We have improved our paper in the revised version based on your suggestions.

Reviewer Comment 1.2 —

There is another way to compress big DL model called model quantization/pruning. As is known, each parameter is stored in a 32-bit data structure. You can simply quantize/prune the model using the state-of-the-art techniques and compare them with knowledge distillation. It would increase the popularity of the paper.

Response:

As the comment suggested, we have performed new experiments by quantizing the parameters of our model using quantization-aware training proposed in [85]. New results are reported in Table 8 (page 17) and discussed under subsection 'Model Compression using Quantization' (page 17).

In Table 8 (Page 17):

Table 8: Comparison among quantization and KD methods for model compression. Experiments are done on MobileNetV2 architecture on the CIFAR 10 dataset. KD techniques use ResNet18 as the teacher. `Ours` method achieves the best performance in both accuracy and carbon footprint metrics.

Method	Accuracy (%) ↑	GFLOPs (M) ↓	Energy (kWh) ↓	CO_2_eq (g) ↓
No compression/KD	90.52	2.16	0.16	45.02
Quantization[85]	89.97	**2.46**	0.40	124.42
KD[26]	91.62	128.30	3.99	1173.44
`Ours`	**91.78**	6.42	**0.21**	**61.76**

In Page 17:

Model Compression using Quantization

In addition to KD, model quantization can be another way of model compression. In Table 8, we compare a quantization method named quantization-aware-training [85] with the KD methods used in this paper. Deep learning models store parameters as floating points (32 bits or 64 bits) to achieve high precision and accuracy. However, quantization-based methods quantize the precision of input and parameters by reducing bit-width to integers (8-bit), reducing the model size, inference time, and performance. Table 8 showcases that the KD technique consumes 3.99kWh of energy and produces 1173.44g of CO_2, and performs with an accuracy of 91.62%. The MobileNetV2 model produced by quantization-aware training builds a smaller and faster model but lacks performance with an accuracy of 89.97%. The quantized model consumes significantly lower energy as opposed to KD. Nonetheless, our proposed stochastic technique consumes lower power (0.21kWh), produces a smaller carbon footprint overall, and performs similarly to both methods.

Reviewer Comment 1.3 —
There are no sample images so it is very hard to interpret results. You can extend the paper with the results and compare in terms of human vision.

Response:
We have added Fig. 4 (Page 14) including sample images and visual results. The related discuss is in subsection 'Main Results' (Page 15).

In Page 15:
In Figure 4, we further visualize the output of No compression, KD [26], and `Ours` methods. The output logit shown in Figure 4(a) indicates that all methods have correctly classified a sample bird image input because the class bird gets the highest score. Figure 4(b) showcases Grad-Cam images from the last layer of the MobileNetV2 architecture. `Ours` and KD [26] methods produced similar Grad-cam visualization, suggesting that both have learned from the same teacher. High values at the diagonal positions of the confusion matrices in Figure 4(c) demonstrate that `Ours` method is equally successful with No compression and KD [26] methods.

In Figure 4 (Page 14)

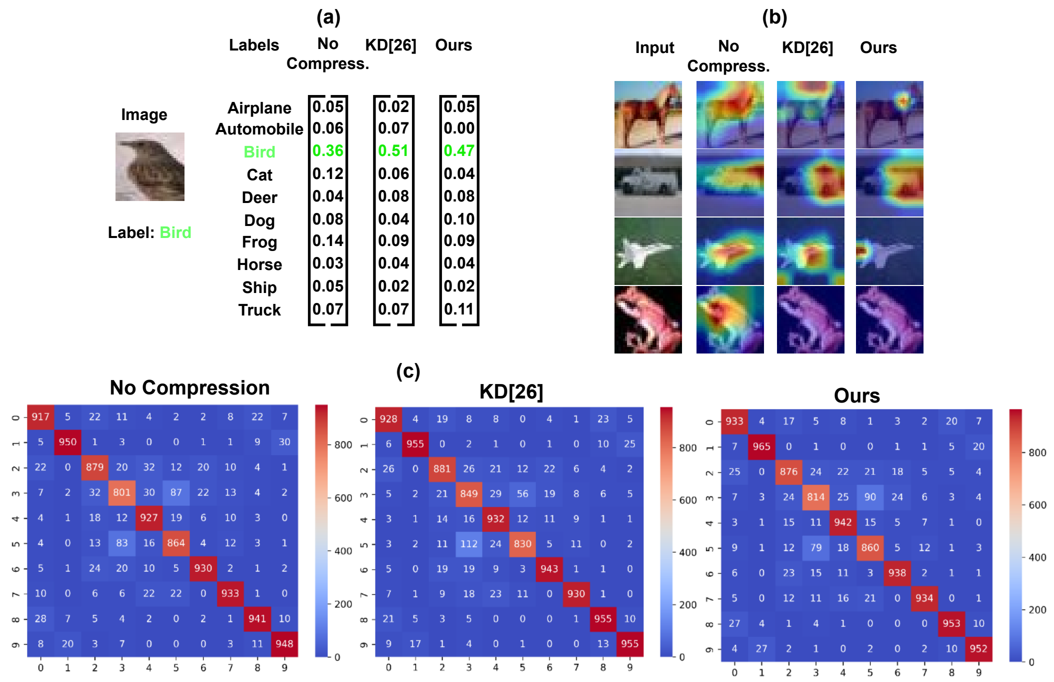

Figure 4: Visual illustration of output produced by No compression, KD [26] and `Ours` methods. (a) Output logits for a sample 'bird' image from the CIFAR 10. (b) Grad-Cam visualization of four sample images from the CIFAR 10 using the MobileNetV2. ResNet18 is used as. (c) Confusion matrices of prediction.

Reviewer 2

Reviewer Comment 2.1 —

The authors examined the environmental costs (carbon footprints) for deep learning model compression using knowledge distillation. The authors have extensively experimented with different combinations of student-teacher models based on the architectures of ResNet18, MobileNetV2 and ShuffleNetV2, as well as CIFAR10, CIFAR 100, Tiny Imagenet and PASCAL VOC reported both object recognition and detection problems using their datasets. Researchers have taken up a very interesting study indeed. My reviews and suggestions about their publications are listed;

Response:
We appreciate the encouraging comments. We have revised the paper in accordance with your insightful and constructive comments. Thank you.

Reviewer Comment 2.2 —

More numeric values should be given in the abstract. The abstract should include the context or background information for your research; the general topic under study; the specific topic of your research;

why is it important to address these questions; the significance or implications of your findings or arguments. It must also contain more numeric values. Please highlight your contribution. Reorganize the abstract to conclude: (a) The overall purpose of the study and the research problems you investigated. (b) The basic design of the study. (c) Major findings or trends found as a result of the study. (d) A brief summary of your interpretations and conclusions.

Response:
Thank you for the suggestion. We have revised the abstract based on the review's suggestion.

In Page 1:

Abstract

Deep learning techniques have recently demonstrated remarkable success in numerous domains. Typically, the success of these deep learning models is measured in terms of performance metrics such as accuracy and mean average precision (mAP). Generally, a model's high performance is highly valued, but it frequently comes at the expense of substantial energy costs and carbon footprint emissions during the model building step. Massive emission of CO_2 has a deleterious impact on life on earth in general and is a serious ethical concern that is largely ignored in deep learning research. In this article, we mainly focus on environmental costs and the means of mitigating carbon footprints in deep learning models, with a particular focus on models created using knowledge distillation (KD). Deep learning models typically contain a large number of parameters, resulting in a 'heavy' model. A heavy model scores high on performance metrics but is incompatible with mobile and edge computing devices. Model compression techniques such as knowledge distillation enable the creation of lightweight, deployable models for these low-resource devices. KD generates lighter models and typically performs with slightly less accuracy than the heavier teacher model (model accuracy by the teacher model on CIFAR 10, CIFAR 100, and TinyImageNet is 95.04%, 76.03%, and 63.39%; model accuracy by KD is 91.78%, 69.7%, and 60.49%). Although the distillation process makes models deployable on low-resource devices, they were found to consume an exorbitant amount of energy and have a substantial carbon footprint (15.8, 17.9, and 13.5 times more carbon compared to the corresponding teacher model). The enormous environmental cost is primarily attributable to the tuning of the hyperparameter, Temperature (τ). In this article, we propose measuring the environmental costs of deep learning work (in terms of GFLOPS in millions, energy consumption in kWh, and CO_2 equivalent in grams). In order to create lightweight models with low environmental costs, we propose a straightforward yet effective method for selecting a hyperparameter (τ) using a stochastic approach for each training batch fed into the models. We applied knowledge distillation (including its data-free variant) to problems involving image classification and object detection. To evaluate the robustness of our method, we ran experiments on various datasets (CIFAR 10, CIFAR 100, Tiny ImageNet, and PASCAL VOC) and models (ResNet18, MobileNetV2, Wrn-40-2). Our novel approach reduces the environmental costs by a large margin by eliminating the requirement of expensive hyperparameter tuning without sacrificing performance. Empirical results on the CIFAR 10 dataset show that the stochastic technique achieves an accuracy of 91.67%, whereas tuning achieves an accuracy of 91.78% - however, the stochastic approach reduces the energy consumption and CO_2 equivalent each by a

factor of 19. Similar results have been obtained with CIFAR 100 and TinyImageNet dataset. This pattern is also observed in object detection classification on the PASCAL VOC dataset, where the tuning technique performs similarly to the stochastic technique, with a difference of 0.03% mAP favoring the stochastic technique while reducing the energy consumptions and CO_2 emission each by a factor of 18.5.

Reviewer Comment 2.3 —

Some table and figure texts are really long. These need to be shortened. Figure texts and main texts should be separated from each other.

Response:
In the revised manuscript, we have shortened the captions of Figures 1, 2, and 3, Tables 1 and 4.

Reviewer Comment 2.4 —

The mathematical background for the proposed method presented in the "Stochastic solution" section should be presented.

Response:
We have added the mathematical underpinning of our method under the subsection 'Stochastic solution' (Page 9). It shows that our proposed stochastic τ brings a regularizing effect in soft logits which helps to eliminate the need for validated τ during the KD process.

In Page 9:
Considering a sample input with its label, $(\boldsymbol{X}, y)$ which is an element in dataset D, a student model $\mathcal{F}_s(\boldsymbol{X}, \boldsymbol{W_s})$ produces soft logits $\boldsymbol{o_s}$ from unactivated logits $\boldsymbol{a_s}$. The training objective is to match the soft logits $\boldsymbol{o_s}$ to the soft logits $\boldsymbol{o_m}$, of a large teacher $\mathcal{F}_m(\boldsymbol{X}, \boldsymbol{W_m})$, from the teacher's own unactivated logits $\boldsymbol{a_m}$, softened by a temperature τ, using an altered softmax $\boldsymbol{o_z} = \sigma(\boldsymbol{a_z}) = \frac{e^{\boldsymbol{a_z}(i)/\tau}}{\sum_{j=1}^{n} e^{\boldsymbol{a_z}(j)/\tau}}$ where, $\boldsymbol{a_z}(i), \boldsymbol{a_z}(j) \in \boldsymbol{a_z} \subset \mathbb{R},\ |\boldsymbol{a_z}| = n,\ 1 <= i, j <= n$. Knowledge distillation uses a KL divergence loss as additional information from the teacher given by $\mathcal{L}_{KLD}(\boldsymbol{o_s}, \boldsymbol{o_m}) = \tau^2 \sum_{c=1}^{n} \boldsymbol{o_m}(c) \log \frac{\boldsymbol{o_s}(c)}{\boldsymbol{o_m}(c)}$ (as shown in Eq 1) softened and weighted by τ. Considering the model is learning for ω epochs and with a constant τ as in traditional KD, the teacher output $\boldsymbol{o_m}$, remains the same but the student model output $\boldsymbol{o_s}$ changes to optimize towards $\mathcal{L}_{KLD}(\boldsymbol{o_s}, \boldsymbol{o_m})[\omega_p] < \mathcal{L}_{KLD}(\boldsymbol{o_s}, \boldsymbol{o_m})[\omega_q]$, such that $\omega_p > \omega_q$. In this setting, for the input $\boldsymbol{X}$ the target $\boldsymbol{o_m}$ is constant throughout the training cycle of the student due to a fixed τ. In our proposed stochastic approach, the value of τ is varied stochastically for every batch, thus ensuring that the target $\boldsymbol{o_m}$ for an input sample $\boldsymbol{X}$ remains varies. This variety in $\boldsymbol{o_m}$ may prevent $\mathcal{F}_s(\boldsymbol{X}, \boldsymbol{W_s})$ from learning to optimize from a single $\boldsymbol{o_m}$ and therefore likely providing a regularizing effect. This may eliminate the need to search for a fixed validated τ.

Reviewer Comment 2.5 —

Although some evaluation criteria are given in the article, It should be well supported by Precision, Recall (sensitivity), Specificity, Prevalence, Kappa, and F1-score. These results need to be analyzed, tabulated, presented graphically, and interpreted.

Response:
As suggested, we have reported Precision, Recall (sensitivity), Specificity, Prevalence, Kappa, and F1-score for ResNet18 as a teacher, MobileNetV2 as a student using CIFAR 10 dataset. Results are reported in Table 5 (Page 13). It is worth noting here that the main focus of this paper is to estimate the environmental cost of the Knowledge distillation process rather than improving model performance. Therefore, we report carbon footprints (FLOPs count in millions, energy consumption in kWh, and CO_2 equivalent in grams) for all experiments. In contrast, we report Precision, Recall, Specificity, etc., for one student-teacher case.

In Table 5 (Page 13):

Table 5: Different performance metrics of ResNet18-MobileNetV2 model combination on the CIFAR 10 dataset.

Model	Accuracy	Precision	Recall	Specificity	Kappa	Prevalence	F1
Teacher	94.80	0.95	0.95	0.95	0.94	0.1	0.95
KD [26]	91.67	0.92	0.92	0.92	0.90	0.1	0.92
`Ours`	91.78	0.92	0.92	0.92	0.91	0.1	0.92

In Page 15:

In Table 5, we further report Precision, Recall, Kappa, Specificity, Prevalence, and F1 Score for ResNet18-MobileNetV2 teacher-student combination on the CIFAR 10 dataset. The teacher model has an accuracy of 94.8% and produces average precision and recall of 0.95, exhibiting that the models have learned all the classes properly. Similarly, the student models achieve nearly equal recall and precision (0.92), corresponding to the accuracy of 91.67% (Tuned) and 91.78% (Ours). We also compute the F1 score which is the harmonic mean of precision and recall. Here, F1 scores of the Teacher model (0.95), KD model (0.92), and Our model (0.92) effectively show that the training is not biased. The Kappa score of our model (0.91) also establishes that the performances and outputs produced are highly agreeable with the labels of the dataset.

Reviewer Comment 2.6 —

Rewrite the conclusion with following comment: (a) Highlight your analysis and reflect only the important points for the whole paper. (b) Mention the implication in the last of this section. Please, carefully review the manuscript to resolve these issues. (c) This section should be supported with numerical values. You should use a more academic language. The authors should polish the manuscript to improve its writing. The quality of the article should be increased. English must be strongly revised to make the paper even readable.

Response:
Thank you for the suggestion. We have rewritten the Section Conclusion based on the review's suggestion.

In Page 17:

Conclusion

The deployment of large models is infeasible to mobile and edge computing devices. Knowledge distillation provides a solution to this issue by generating a significantly lighter and deployable model on mobile and edge computing devices. However, we demonstrate that this suitability often comes at the expense of substantial environmental costs, largely ignored in deep learning literature. The objective of this article is threefold: (1) to investigate environmental costs for deep learning model compression using knowledge distillation, (2) to propose a stochastic approach as a means to mitigate carbon footprints for the knowledge distillation process, and (3) to conduct extensive experiments that demonstrate the suitability of the proposed stochastic approach. We propose that deep learning research should not be measured only by performance metrics such as accuracy and mean mAP, rather the performance metrics should also include environmental costs. Extensive experiments were conducted using various student-teacher model combinations (based on ResNet18, MobileNetV2, and ShuffleNetV2) to solve image classification and object detection problems. We estimated the carbon footprints of the overall computation process for each student-teacher model combination in terms of FLOPs count in millions, energy consumption in kWh, and CO2 equivalent in grams. Based on our empirical findings, KD consumes 13 to 18 times more carbon than the heavier teacher model. The repetitive tuning of hyperparameters is primarily responsible for such astronomical environmental costs. This article investigates the environmental impact of model compression techniques based on knowledge distillation and proposes a stochastic approach that requires less computation without sacrificing performance. Empirical results demonstrate that the proposed stochastic approach applied on CIFAR 100 dataset consumes 0.20kWh of energy and emits 61.60g of CO_2 while the tuning approach consumes 3.80kWh of energy and emits 1170.40g of CO2 with a performance difference favoring the tuning approach of 0.30%. Similarly, for the Tiny ImageNet dataset, the performance accuracy of the stochastic approach is 60.53 percent, compared to tuning's performance accuracy of 60.46 percent. However, the stochastic technique only consumes 1.04kWh of energy and emits 304.88g of CO2, whereas tuning approach emits a massive amount of carbon footprint (5,595.31g) and uses a tremendous amount of energy (19.09kWh). In future, we intend to investigate the carbon footprint of additional deep-learning tasks, such as continuous learning, domain adaptation, and meta-learning, in which the same model undergoes multiple training rounds.
